# Morphological changes in the lower Lancang River due to extensive human activities

**DOI:** 10.7717/peerj.9471

**Published:** 2020-07-23

**Authors:** Peijia Wang, Kaidao Fu, Jiangcheng Huang, Xingwu Duan, Zaizhi Yang

**Affiliations:** 1 Institute of International Rivers and Eco-Security, Yunnan University, Kunming, Yunnan, China; 2 Yunnan Key Laboratory for International Rivers and Transboundary Eco-Security, Yunnan University, Kunming, Yunnan, China

**Keywords:** Mountain river, Lancang-Mekong, Multi-source remote sensing image, River morphological changes, Influencing factors

## Abstract

**Background:**

Morphological changes in river beds determine the risk for soil erosion within floodplain areas. At present, little is known about river morphological changes in high-altitude mountainous area influenced by extensive human activities. The study has important reference value for clarifying the morphological changes of mountainous rivers due to extensive human activities.

**Methods:**

Based on the long-term multi-source remote sensing image data, we analyzed morphological changes in the lower Lancang River (LCR) in Southwest China at regional and local scales, and determined their influencing factors. Visual interpretation is used to extract the information and unify the interpretation standards of various localities, mainly including water bodies, sandbars, shorelines and beaches. Based on high-resolution remote sensing images, we analyzed the planforms, erosion and accretion conditions in Jinghongba (JHB) reach and Ganlanba (GLB) reach.

**Results:**

Our results showed that: (1) during 1993–2016, the planforms of Jinghongba and Ganlanba in the wide valley of the lower LCR changed significantly, (2) In the interval 1993 to 2016, the JHB reach exhibited extensive erosion, with the right bank more eroded seriously than the left bank, and an erosion area of 0.36 km^2^. The erosion area of the GLB reach was 0.33 km^2^, with accretion area of 0.61 km^2^. The right bank was dominated by erosion, while the left bank by sedimentation; (3) Morphological changes in JHB and GLB reches were the result of the combined effects of cascade hydropower development, river regulation, and sand-mining in the river. The results improved the understanding of riverbed evolution, and could help guide sediment management in mountainous areas influenced by extensive human activities.

## Introduction

The morphology of natural rivers is determined by the long-term interaction of water, sediment, and riverbed conditions (*Fu & He, 2007*). Certain flow conditions (flow rate, viscosity), riverbed conditions (shape, slope, roughness, and sediment composition), and boundary conditions (water surface width, and water depth) play significant roles in the evolution of river morphology (Gupta & Liew, 2007). Besides, intensification of human activities further affects the evolution processes of riverbed morphology (*Church, 2002*).

Studies on river morphological changes included mainly changes in water amounts and sediment loads (Singha & Balachandar, 2011; Wang et al., 2016), lateral changes in river channels (Tamura et al., 2010; Turki et al., 2013; Truong, Ye & Stive, 2017), longitudinal deformation (Williams & Wolman, 1984; Dade, 2000; Ralph & Hesse, 2010), micro-geomorphic evolution of river channels (*Ronco et al., 2010; Eaton, Millar & Davidsons, 2014*), the relationship between water and sediment variability, and river bed evolution (*Xia et al., 2014*). Many of these researches focused on the lower reaches of rivers and deltas in plains, and focused on rivers such as the Rhine, the Amazon, the Nile River Delta (*Benn & Erskine, 1994; Ralph & Hesse, 2010; Lee et al., 2016*), the Yellow River (Jiang, Pan & Chen, 2017), and the Yangtze River (Xue, Liu & Ge, 2011; Xia et al., 2017). However, morphological changes in high-altitude rivers are still less explored, and mainly concentrated in the mountainous areas of Europe (*Scorpio et al., 2016; Kidová, Lehotský & Rusnák, 2016; Hanna & Wyzga, 2019*). However, applications of remote sensing images in the analyses of morphological changes in mountainous river are scarce (Scorpio & Rosskopf, 2016).

The Lancang-Mekong is an important international river in Asia (*Liu, Trinder & Turner, 2017*). At present, researches on changes in river morphology in this basin are mainly concentrated in the Mekong Delta region, and includes sediment transport, Shoreline undulations and monitoring of river channel erosion and accretion (Parker, 1978; Milliman & Syvitski, 1992; Rüther & Olsen, 2003; Rüther & Olsen, 2007; Phan, Van Thiel de Vries & Stive, 2015; Brunier et al., 2014; Paiement Paradis, Marquis & Roy, 2011). Researches in the lower Lancang River (LCR) Basin in China are mainly focused on the assessment of single factor index, such as the runoff and sediment load (Huang, Fu & He, 2010; Fu et al., 2015), with less attention on river morphology and sediment accretion. Hydropower development and sand excavation activities are concentrated in the lower reaches of LCR, Many factors influence sediment transport, erosion and accretion processes of rivers in mountainous areas (*Li et al., 2017*). On the one hand, they are related to the hydrodynamic characteristics of the river, that is, water inflow and sediment conditions (*Rodríguez-Blanco, Taboada-Castro & Taboada-Castro, 2010; Tuset, Vericat & Batalla, 2016*). On the other hand, they are closely related to the composition of river banks and riverbed. In recent history, climate change and human activities, especially the construction of cascade power stations, land use and sand-mining, changed the runoff-sediment relationship of rivers, which impact on erosion and sedimentation process (*Cochrane, Arias & Piman, 2014; Zhai et al., 2016; Nguyen et al., 2018*). but little work has combined the influencing factors of dams, river-bed mining, environmental protection (*Zhai et al., 2016*).

The middle and lower reaches of the LCR is important hydropower energy bases in China due to the vast hydropower resource (*Li et al., 2017*). Since the 1990s, large-scale cascade hydropower development in this region, coupled with extensive human activities, has made significant changes in the inflow of water and sediment in the lower LCR (*Fu et al., 2015*). However, the mechanism of the variation of the downstream hydrological process and the response of the river system to the interference of extensive activities is still unclear. To improve the understanding of the alteration process in lower LCR, we analyzed morphological changes for regional scale of Jinghong to Guanlei reach, local scales of Jinghongba (JHB) and Ganlanba (GLB) reaches using multi-source remote sensing images for years 1993 to 2016. The possible influencing factors of river morphology change were also discussed in this paper.

## Data and Methods

### Study area

**Figure 1:**
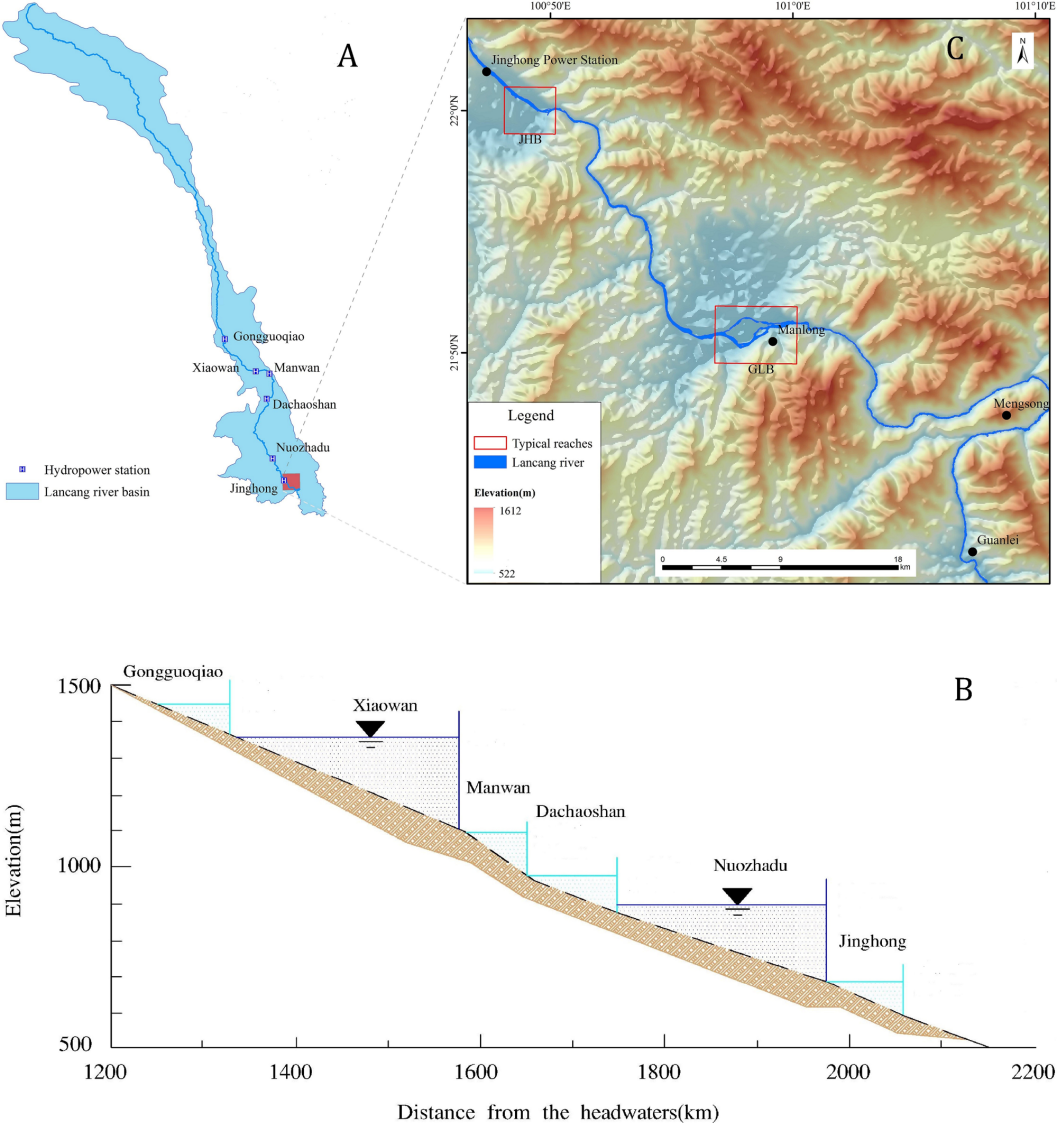
(A) Location of dams in LCR main stream basin, China; (B) Parameters of cascade power station in the middle and lower reaches of LCR; (C) Location of the study area. The map of the LCR basin in (A) and the Digital Elevation Model (DEM) in (C) were provided by the Data Center for Resources and Environmental Sciences, Chinese Academy of Science (http://www.resdc.cn/). Parameters of cascade power station in the middle and lower reaches of LCR in (B) was obtained from HydroChina, 2010, Map of planned and existing hydropower projects (http://www.hydrochina.com.cn/).

LCR originates at the northern foot of Tanggula Mountains on the Qinghai-Tibet Plateau and flows southeast through Qinghai, Tibet, and Yunnan, with a total length of 2,161 km, a basin area of 16. 7 ×10^4^ km^2^, a relative height difference of more than 5,000 m, and an average annual runoff of 7. 6 ×10^10^m^3^ (*Fu, He & Li, 2006*) (Fig. 1A). Six cascade hydropower stations have been built on the middle and lower reaches of LCR between 1993 and 2016, with an installed capacity of 1,600 MW (*Li et al., 2017*) (Fig. 1B); among these, Xiaowan and Nuozhadu stations also regulate the annual runoff in addition to power generation (Feng, Mao & Li, 2012).

The lower reaches of LCR between the towns of Jinghong and Guanlei have a natural drop of 253 m *(Fu & He, 2007)*. The valley is 350–600 m wide, and makes a significant turn from the south to the north and southeast (Fig. 1C). The area is characterized by low to medium elevation mountains, and JHB and GLB are located in this region (Fig. 1C). The climate has distinct dry and wet seasons, occurring from November-April and May–October, respectively *(Zuo, Liu & Qian, 2011)*. In most areas, annual precipitation exceeds 1000 mm, and 85% of the precipitation is concentrated in wet season (Manh, Merz & Apel, 2013).

### Data

Our data sources mainly include two parts: one is from high-resolution remote sensing images the other is from medium-resolution remote sensing images. Analysis of regional river morphological changes was performed with two medium-resolution remote images, which were Landsat TM of 27 March 1993 and Landsat OLI of 15 February 2016, the two images allowed for identification of river reaches exhibiting significant morphological changes. The process of river morphology changes was further analyzed with high-resolution remote sensing images for two typical reaches, which were called Jinghongba and Ganlanba reaches (Fig. 1C). In JHB reach, QuickBird image was acquired on 19 April 2003 with a panchromatic resolution of 0.61m panchromatic resolution, while Worldview-2 images were acquired on 13 April 2009 and 15 April 2016 with a panchromatic resolution of 2.5 m. In Ganlanba reach, IKonos image on 19 February, Worldview-2 image on 14 February 2009 and GF-2 image on 2 February 2016 were chosen to map the river channels (Table 1).

**Table 1:** List of remote sensing image data.

**Coverage area**	**Satellite sensor**	**Acquired dates of images**	**PanchromaticResolution(m)**	**Multispectral** (m)
Jinghong to Guanlei reach	Landsat TM	1993-3-27	/	30
	Landsat OLI	2016-2-15	15	30
JHB	QuickBird	2003-4-19	0.61	2.44
	Worldview-2	2009-4-13	0.5	2
	Worldview-2	2016-4-15	0.5	2
GLB	IKonos	2003-2-19	1	4
	Worldview-2	2009-2-14	0.5	2
	GF-2	2016-2-9	0.8	3.2

Acquired date difference of remote sensing images will have a great impact on the analysis of river morphological changes (*Manh, Merz & Apel, 2013; Zhao et al., 2013; Shrestha et al., 2016*). In this study, the influence of water level on the results was diminished using three methods. First, the remote images from February to April (dry season) were used to maintain the consistency of images acquisition dates as much as possible. Second, the width of water surface for a typical reach at multiple periods was calculated to make the width of water surface of selected image data less than 0.1m. Finally, by comparing water levels measured at two neighboring hydrological stations, which called Jinghong Hydrological Station at upstream and GLB Hydrological Station at downstream, the corresponding water level variability in the observation reaches at multiple periods was determined to be less than 0.5 m.

The construction of hydropower stations has changed the natural hydrological process in the lower reaches of the LCR (Fan, He & Wang, 2015), so considering the water storage time at the cascade power stations in LCR, the 1993 and 2016 Landsat images were selected as data sources for the regional scale of Jinghong to Guanlei reach. Additionally, we selected high-resolution images for JHB and GLB, the two wide-valley reaches of LCR (Table 1).

### Data processing of remote sensing images

At regional and local scales, we took the high-resolution and medium-resolution remote sensing images as the data source respectively. Eight remote sensing images were preprocessed through band composition, geometric correction, image enhancement and other data in ENVI software. We established the interpretation marks of the eight remote sensing images according to the data and the spectral characteristics of the objects.

### Extraction method and interpretation of river water

As the research object was the change of river information, high accuracy of river boundary was required. We combined computer automatic interpretation, and manual visual interpretation and modification to reach this goal.

According to the spectral characteristics of ground objects, the reflectance difference of water bodies in the green band was not obvious, and there was a large difference in the near-infrared band. By comparison, it was found that only the reflectance of water bodies in the green band is higher than that in the near-infrared band. Therefore, in order to expand the spectral difference between water bodies and other ground objects, the Normalized Difference Water Index (NDWI) was obtained. For Landsat TM and OLI images, Green and NIR were band 3 and band 5, respectively. NDWI could effectively suppress vegetation information, but the effect of suppressing residential areas was not good. On the basis of NDWI, Xu Hanqiu proposed an improved Modified Normalized Difference Water Index (MNDWI).

\begin{eqnarray*}\mathrm{MNDWI}= \frac{\text{Green}-\mathrm{MIR}}{\text{Green}+\mathrm{MIR}} \end{eqnarray*}

where MNDWI was the modified normalized difference water index, MIR was the mid-infrared band, and the fifth band in Landsat TM and OLI. Green was the green band, and the second band in Landsat TM and OLI. We have set the threshold value of MNDWI to 0, extracted the water boundary information, automatically extracted the results by computer and manually modified the images to obtain the river information map from Jinghong to Guanlei reach of lower LCR.Visual interpretation of high-resolution images of typical reaches was used to extract information and unify the interpretation standards of various localities, mainly water bodies, sandbars, shorelines, and point bars. Based on high-resolution images of two typical reaches of the Jinghong to Guanlei reach of LCR, we analyze the planforms, and erosion and sedimentation conditions, and determined changes of the two river reaches during 1993 to 2016.

### Parameter data of river planforms

**Figure 2:**
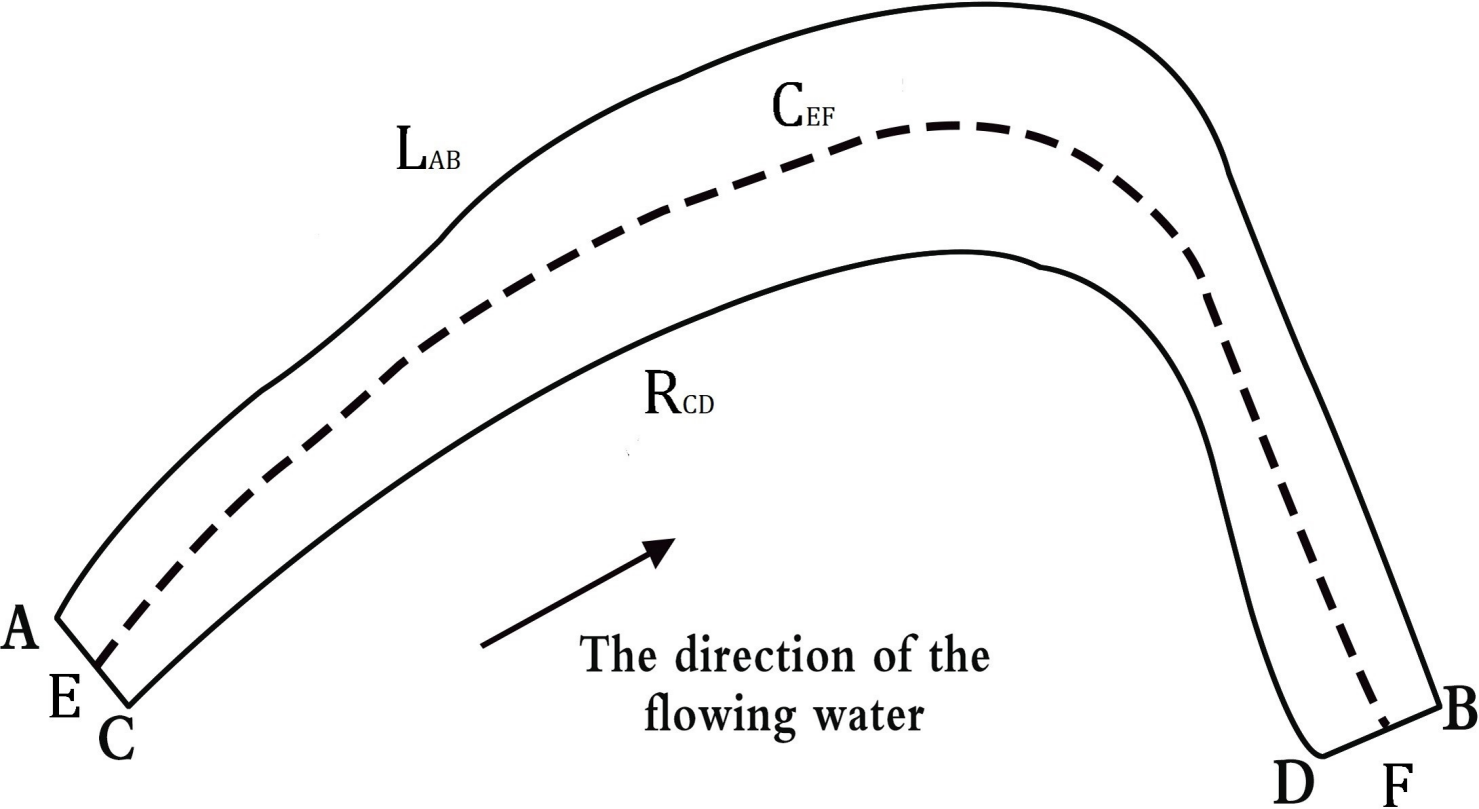
Definition of plane configuration parameters.

The river planforms are important features of river geomorphology (*Kummu et al., 2008*). According to the topographic and geographical characters of the study area, five parameters were selected as indicators: river area (*S*_*ABCD*_), average river width (*A*_*w*_), left bank length (*L*_*AB*_), right bank length (*R*_*CD*_), center line length (*C*_*EF*_). Spatial analysis and parameter value calculation were completed using GIS 10.4 software. The river area (*S*_*ABCD*_) was the instantaneous water surface area of the river; the average river width (*A*_*W*_) was automatically calculated for every 50-m river interval using ArcGIS software, and then an average river width was obtained; left bank length (*L*_*AB*_) is shown below on the left along the river flow direction; right bank length (*R*_*CD*_) is shown below on the right along the river flow direction (Fig. 2).

The relationships among the changes of five planforms parameters were analyzed in typical reaches of Jinghong to Guanlei reach of LCR. The significance level P obtained by the difference significance test between each two periods reflected the significance of the change of planforms parameters of two typical reaches, If *P* < 0.05, it meaned that there was a significant statistical change during the study period of the channel’s planform parameters. Then we analyzed data using Origin 10.4 and R3.3.3 software to calculate statistical parameters.

## Results

### Characteristics of regional river morphology changes

**Figure 3:**
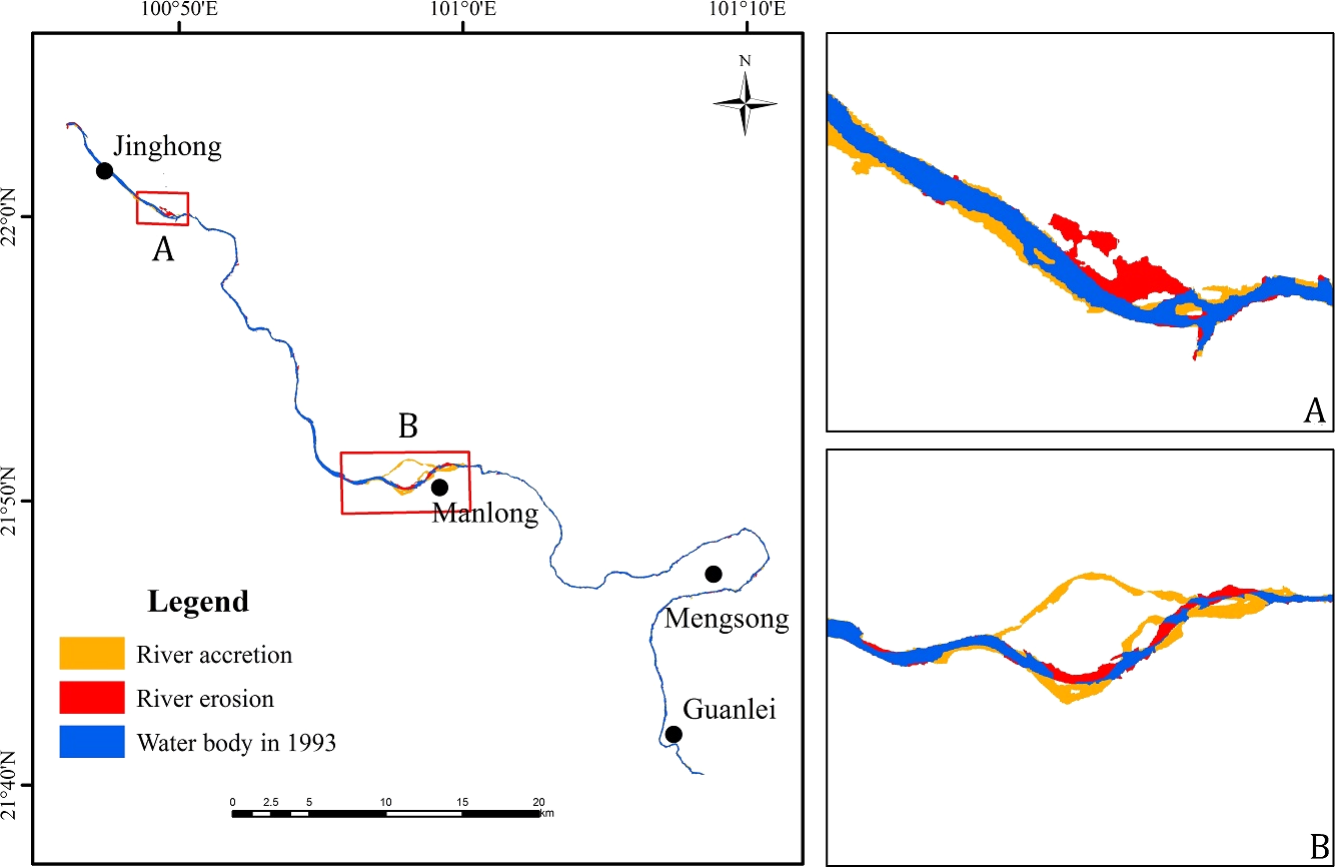
Map of erosion and accretion distribution in the Jinghong to Guanlei reach of LCR between 1993 and 2016. (A) Distribution of erosion and accretion in JHB reach from 1993 to 2016; (B) distribution of erosion and accretion in GLB reach from 1993 to 2016.

**Figure 4:**
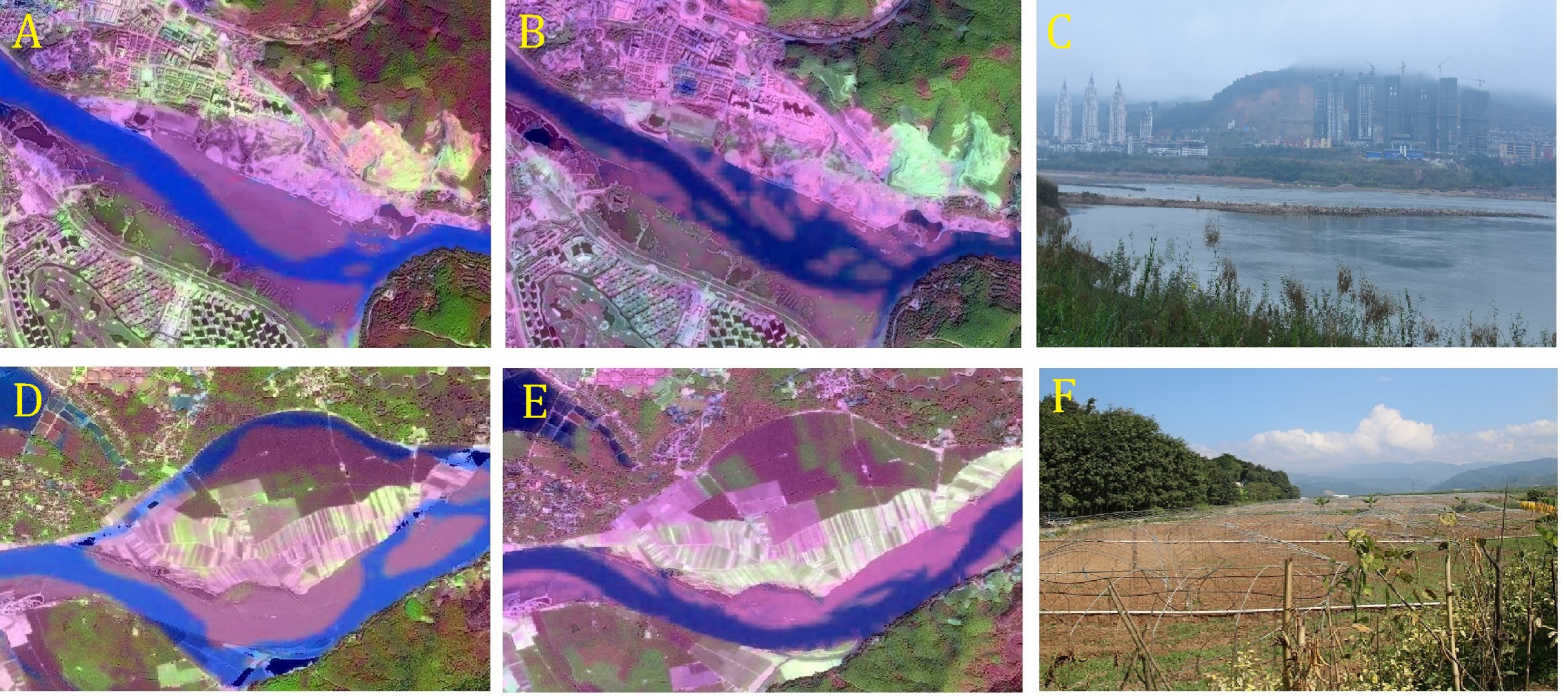
Remote sensing images and photos in Jinghongba reach and Ganlanba reach. (A) Landsat TM image of March 27, 1993 in Jinghongba reach; (B) Landsat OLI image of February 15, 2016 in Jinghongba reach; (C) Photo of December 24, 2018 of field survey in Jinghongba reach; (D) Landsat image of March 27, 1993 in Ganalanba reach; (E) Landsat OLI image of February 15, 2016 in Ganalanba reach; (F) Photo of December 24, 2018 of field survey in Ganlanba reach. Satellite image source (A, B, D, E): USGS, http://glovis.usgs.gov/.

The study at regional scale revealed an increasing trend in erosion area, and a decreasing trend in sedimentation area in the reach of LCR from Jinghong to Guanlei during 1993 to 2016 (Fig. 3). Most of the erosion and sedimentation phenomena were concentrated in specific wide valley reaches, include JHB, GLB, Menglong and Guanlei reaches, of which changed significantly in JHB and GLB reaches. In JHB reach, erosion on the left bank was notable, while accretion on the right bank was obvious; In GLB reach, the bifurcated channel on the left side disappeared due to extensive accretion in the river channel, and slight erosion was observed on the left channel (Fig. 4).

### Morphological changes of typical reaches

#### Change of river planform parameters

**Figure 5:**
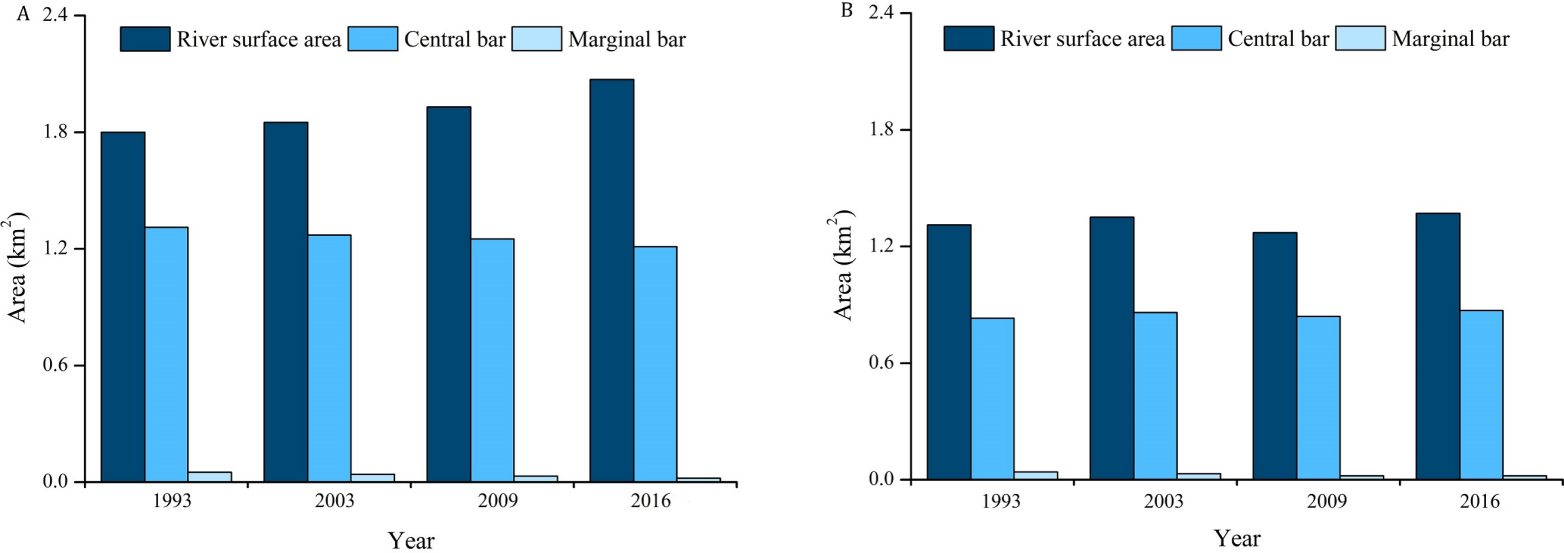
JHB and GLB reaches Surface Statistics from 1993–2016. (A) Surface statistics of JHB reach from 1993–2016; (B) Surface Statistics of GLB reach from 1993–2016.

**Figure 6:**
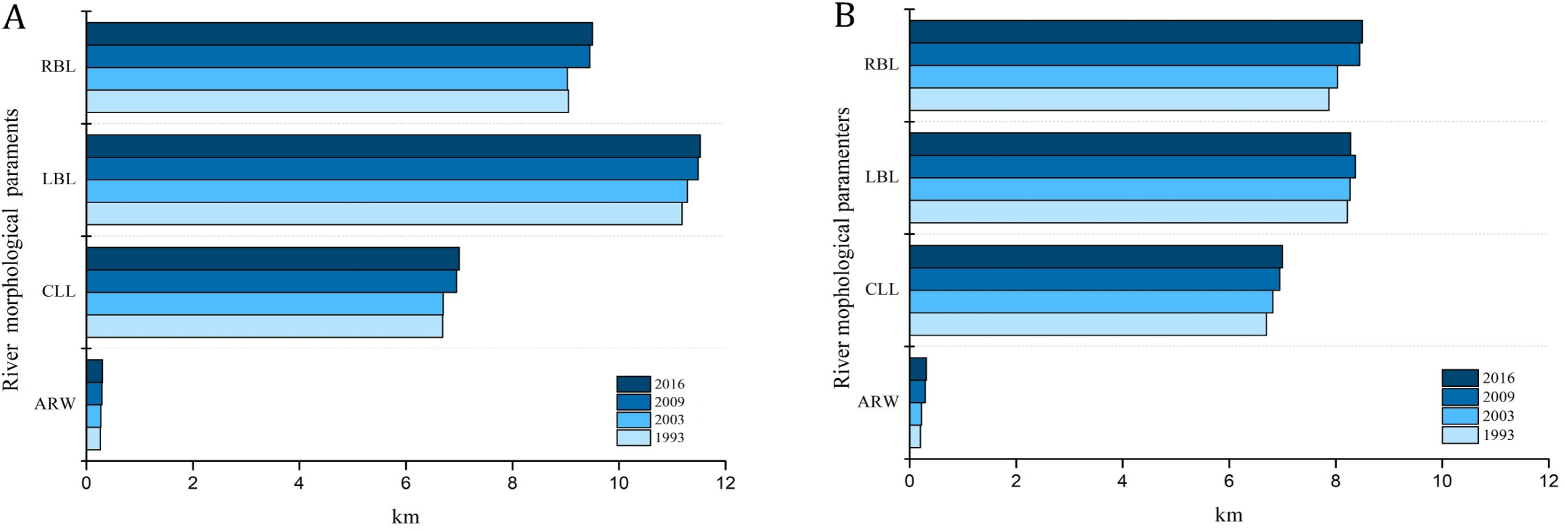
JHB and GLB River planforms from 1993–2016. (A) River planforms of JHB from 1993–2016; (B) River planforms of GLB from 1993–2016. (CLL: Center line length; LBL: Left bank length; RBL: Right bank length; ARW: Average river width).

Our results showed that GLB and JHB reaches experienced significant changes in the river planforms. In JHB reach, the main river area exhibited an increasing trend from 1993 to 2016, with the most noticeable growth from 2003 to 2009. The areas of the central and the marginal bars decreased by 0.1 km^2^ and 0.03 km^2^, respectively (Fig. 5A). In GLB reach, the main channel area has increased by 0.09 km^2^ from 1993 to 2016, while the areas of central bar and the marginal bar have decreased respectively by 0.03 km^2^ and 0.04 km^2^ (Fig. 5B). The average river width of JHB and GLB followed the same trend as the area of the main channel, and both were increasing, and the most obvious changes occurred from 2003 to 2009, with the increasing rate as high as 9.3% and 8.1% respectively (Fig. 6A). The length of the left bank, the right bank, and the center line of JHB and GLB increased by varying degrees (Fig. 6B). In that, the length of the left and right bank of JHB and GLB increased by 2.3% and 5.9% respectively, and the variability on the right bank length was significantly higher than that on the left bank. The center line maintained a growth trend, with a relatively large increase of 5.9%.

The trends of river morphological changes in two typical reaches indicated that the area and average river width had an overall growth trend. The length of the left bank, the right bank, and the center line all increased to some degree, while the areas of the central bar and the marginal bar shrunk. The number and size of the relatively stable central bars in 1993 was large. However, the original large-scale central bar has evolved into a small-scale central island. The number of central bars decreased, and the area of the marginal bank also shrunk. JHB and GLB then exhibited erosion and channel widening (Fig. 6).

### Correlation analysis of river planform parameters

**Figure 7:**
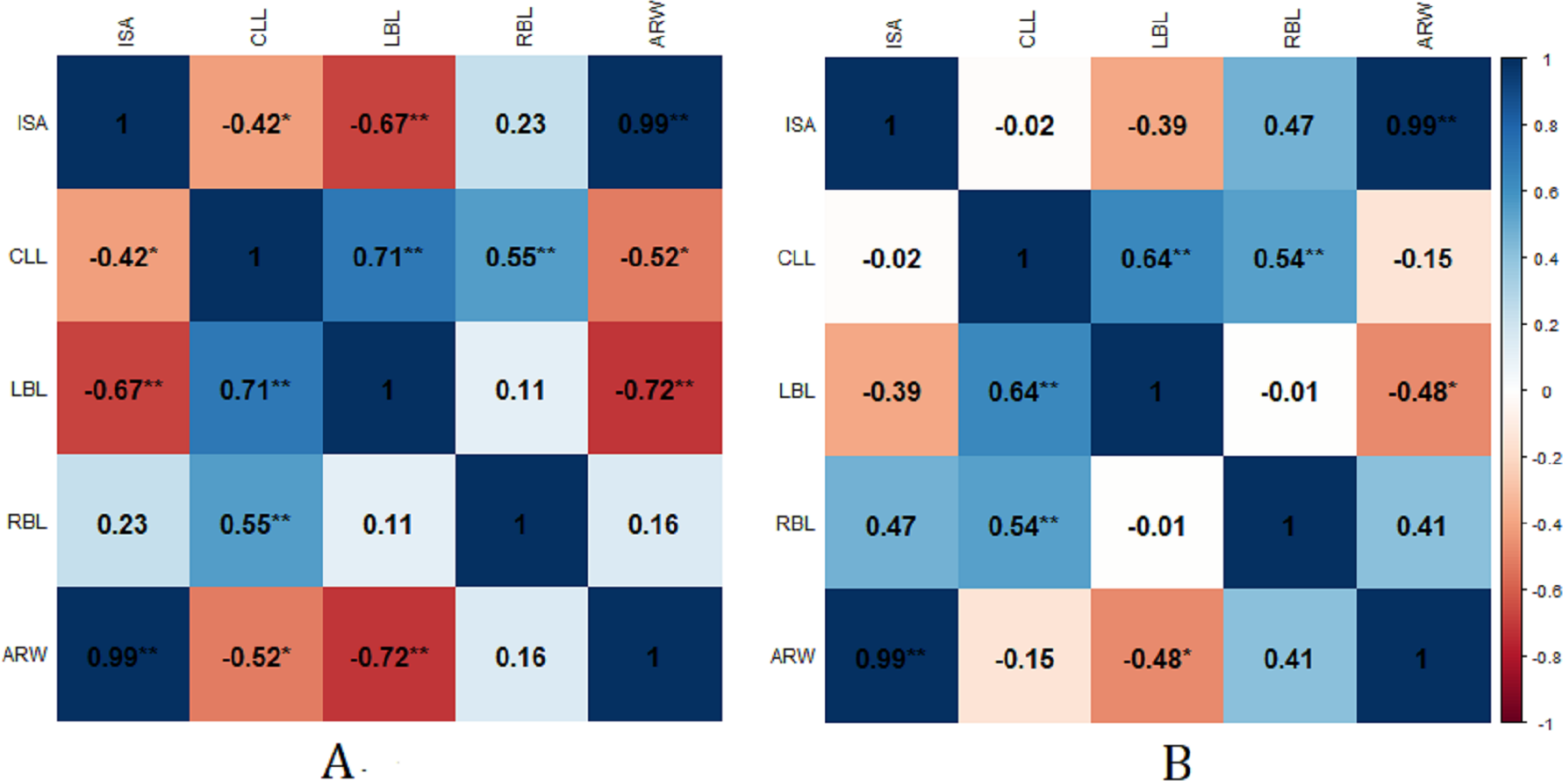
Pearson’s correlation coefficient among environmental parameters in JHB and GLB reach during 1993–2016. (A) Pearson’s correlation coefficient among environmental parameters in JHB reach during 1993–2016. (B) Pearson’s correlation coefficient among environmental parameters in GLB reach during 1993–2016. * *p*-value < 0.05, ^∗∗^*p*-value < 0.01. ISA, Instantaneous surface area; CLL, Center line length; LBL, Left bank length; RBL, Right bank length; ARW, Average river width.

In JHB reach, there was a significant positive correlation between the main channel area and the average river width (*P* < 0.05). The left bank length was significantly correlated with the main river channel area, the average river width, and the center line length, while the right bank length was significantly correlated only with the center line length. The correlation coefficient between the length changes of the left and right banks were extremely small, which indicated that these changes were not directly related in the study area, and that the river banks were changing asynchronously. The main channel area and the average river width of the rivers in GLB had the same significant correlation with JHB. The change in the center line length was significantly related to the length of the left and right banks. This showed that the center line length was sensitive to the changes in erosion and accretion of the left and right banks (Fig. 7).

The main channel area changes in JHB and GLB were positively and highly significantly correlated with the average width changes. The length of the center line was also significantly positively correlated with that of the left and right banks, indicating that the convex bank was covered with sediment and extended, while the concave bank was eroded and extended; the river channel widened, and the center line lengthened.

### Erosion and accretion changes

**Figure 8:**
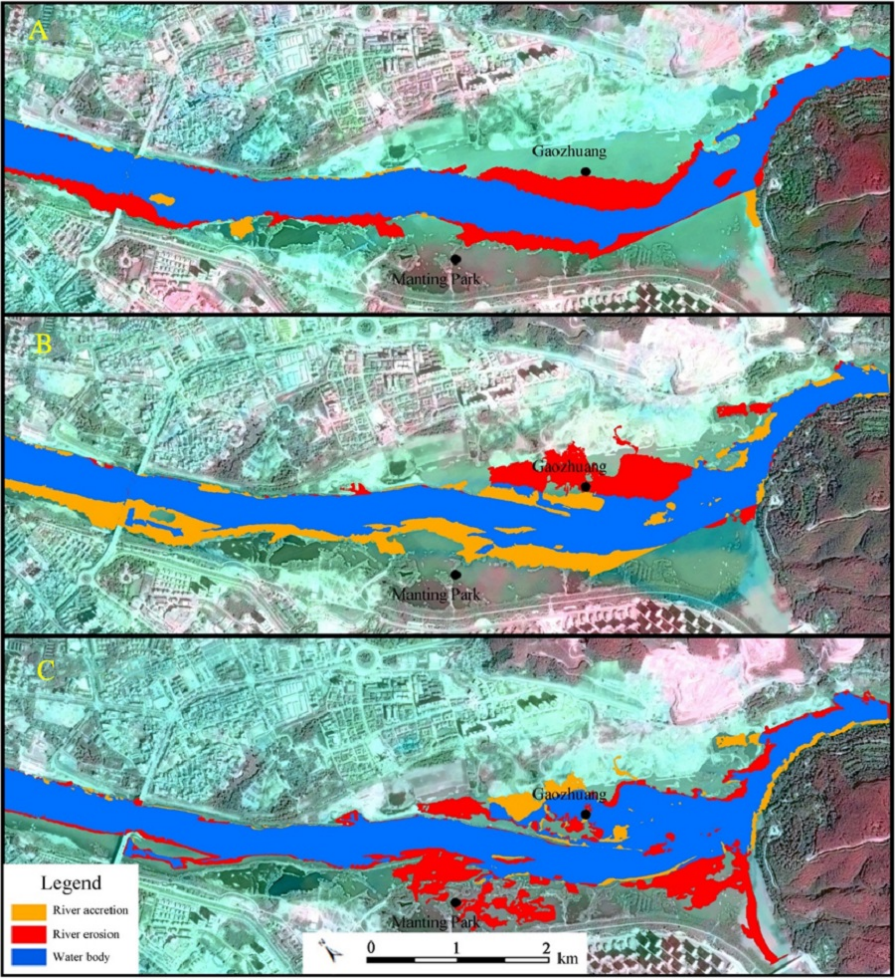
Erosion and accretion in JHB reach from 1993–2016. (A) Erosion and accretion in JHB reach from 1993–2003; (B) Erosion and accretion in JHB reach from 2003–2009; (C) Erosion and accretion in JHB reach from 2009–2016. Satellite image source: Chinese Centre For Resources Satellite Data and Application (http://36.112.130.153:7777/DSSPlatform/).

The JHB reach was dominated by erosion from 1993 to 2003, with the erosion and accretion area were 0.86 km^2^ and 0.02 km^2^ respectively (Fig. 8A). In the interval 2003–2009, JHB exhibited an increasing accretion and decreasing erosion trend. The river on the right was dominated by accretion which continued to increase, while erosion was obvious on the left (Fig. 8B). In the interval 2009–2016, the river channel continued to erode, especially at the Manting Park (Fig. 8C). In the interval 1993–2016, the erosion and accretion areas of JHB were 0.36 km^2^ and 0.21 km^2^ respectively. Erosion was significant in Gaozhuang and Manting Park, and accretion was also well developed on the left bank. Except for the obvious accretion on the right bank during 2003–2009, erosion dominated the rest of the time.

**Figure 9:**
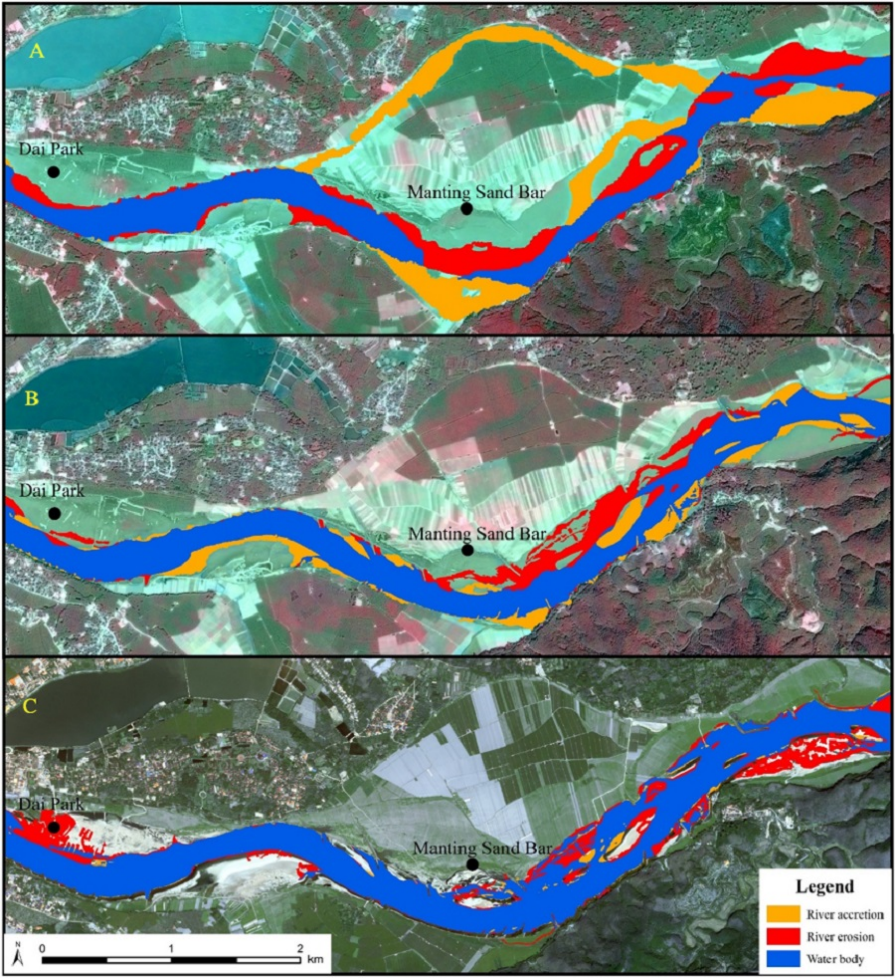
Erosion and accretion in GLB reach from 1993–2016. (A) Erosion and accretion in GLB reach from 1993–2003; (B) Erosion and accretion in GLB reach from 2003–2009; (C) Erosion and accretion in GLB reach from 2009–2016. Satellite image source: Chinese Centre For Resources Satellite Data and Application (http://36.112.130.153:7777/DSSPlatform/).

Accretion on the right bank of GLB occurred mainly from 1993 to 2003, and the accretion and erosion area on the left bank were equivalent at 0.78 km^2^ and 0.75 km^2^, respectively. Particularly the bifurcated channel on the left side disappeared in 1997, which suffered from aggravated accretion (Fig. 9A). During 2003–2009, the erosion area on the left bank of GLB was greater than that of accretion while accretion dominated the right bank of river (Fig. 9B). The main channel of GLB reach became eroded from 2009 to 2016, with an erosion area of 0.38 km^2^. Erosion occurred mostly in the Dai Garden. There was sporadic accretion in Manting Sand Bar, with a accretion area of only 0.08 km^2^ (Fig. 9C). Coexistence of erosion and accretion occurred in the river during 1993–2003, and then experienced a generally decreasing accretion in the period of 2003–2009, after 2009, the river abruptly turned to erosion.

## Discussion

The river morphological change is affected by natural factors and human activities (Zhang et al., 2017; Shrestha et al., 2016; Li et al., 2017). The impact of natural factors on the change of river morphology was relatively slow (Zhao et al., 2013; Gou et al., 2020). In view of the fact that the middle and lower reaches of Lancang River were strongly affected by human activities such as cascade hydropower development, river-bed mining and channel regulation, we only analyzed the river morphological change due to extensive human activities.

### Impact of dam

At present, rivers are generally disturbed by the construction of reservoirs, which not only change the runoff processes, but also have a significant impact on sediment transport rates and load (Xue, Liu & Ge, 2011; Tang et al., 2016). Human activities in the upper reaches of the basin can be sensitively recorded by downstream sediments, and downstream sediment response to hydropower station construction is manifested by increases in sand during construction and reductions after completion (Walling, 2008; Kummu et al., 2010; Fu et al., 2015). About 70% of large river systems across the globe are impounded by man-made reservoirs (Lee et al., 2016); for most rivers, the headwater catchment area is the main source of sediment production and transport, providing more than 75% of the sediment (*Nilsson et al., 2005; Zarfl et al., 2015*). *Fu et al. (2015)* estimated sediment retention ratios of Gongguoqiao, Dachaoshan, and Jinghong reservoirs in the LCR at 60.23%, 66.05%, and 63.50%, respectively. This indicates that construction of cascade dams on LCR has changed the original river connectivity, interrupted sediment transport, changed hydrological conditions and sediment transport process, and resulted in the capture of a sediment portion in the reservoir.

To years 2016, six cascade hydropower stations have been developed in the middle and lower reaches of LCR (*Li et al., 2017*). During 1993–2003 and 2009–2016, the erosion area of JHB was larger than that of accretion. The erosion area of GLB from 2009 to 2016 was also larger than that of accretion. These relationships existed due to the operation of Xiaowan (Total Storage: 151.32 × (10^8^m^3^) and Nuozhadu reservoirs (Total Storage: 223.68 × (10^8^m^3^), which reduce peak flow and increase low flow conditions, and reduce the inter-annual variability in runoff; regulation of flow conditions affects the runoff process of the wide valley reach downstream (Fan, He & Wang, 2015). Additionally, continuity of the river was severed after the Jinghong Hydropower Station started operating in 2008. Sediment concentration was greatly reduced downstream, further changing the rhythm of erosion in wide valley reaches such as JHB and GLB. In addition, sediment grain-size distribution also reflected the dynamic environment of sediment transport and accretion. The cascade hydropower development in the middle and lower reaches of LCR has significantly changed the grain-size composition of sediment, resulting in a significant increase in medium and average grain sizes in the lower reaches of JHB; the increase in median grain size was more notable, the sorting became worse, the skewness tended to be positive, and the peak state was sharpened (Huang, Fu & He, 2010; Fu et al., 2015).

Cascade dams on LCR changed the flow boundary conditions of the upper and lower reaches and reduced sediment discharge, resulting in a significant reduction in sediment load in the lower reaches of the river. In addition, reduction of flood levels triggered an adjustment of the planforms of the riverbed. The change in the runoff sediment relationship caused by cascade hydropower development is the main reason for the change in river morphology in the lower reaches of LCR.

### Impact of channel regulation

A series of channel regulation projects have been carried out over the past decades in the lower reaches of LCR (*Anthony, Brunier & Besset, 2015*). These mainly included: (1) building of 6,594-m long artificial embankment and a riverside green belt from Jinghong station to Gaozhuang in 2008, (2) massive regulation of a 9-km reach of Manting Sand Bar in the 1997 and 2004 respectively (*Zhai et al., 2016*).

Riverbank protection structures influence channel morphology and dynamics by restricting the width of wandering belts (Xu, 1997; Kummu et al., 2008; Skalski et al., 2016). At present, riverbank protection projects are mainly concentrated in the JHB reach. Accretion area in JHB reach was larger than erosion area from 2003 to 2009; this was mainly due to the JHB bank protection projects on both sides of the river channel restricting erosion reach, effectively restraining alteration of the shoreline and the main stream line. In the interval 2009 to 2016, JHB abruptly turned to erosion, reasons mainly included two aspects: one was the riverbank protection structures were safe and stable, and the sedimentation width on both sides of the river channel was limited; the other was the sediment blocking effect of Jinghong hydropower station results in erosion in JHB reach. The river regulation work was mainly concentrated in GLB and lower reaches of LCR. Channel rectification of GLB was carried out from 1993 to 2003, and the branch river on the right side disappeared due to the decrease in water and sediment flow. In addition, in early stages, slow flow, strong carrying capacity for sediment, easily-deposited sediment, and the channel resulted mainly in sedimentation. During the periods of 2003–2009 and 2009–2016, the GLB reach was widened due mainly to river regulation in 1997, 2004, and 2009, the flood level of GLB decreased, and the flood discharge capacity was enhanced. The reach gradually stabilized, and, combined with the regulation effect of cascade dams, water volume in the dry season increased, and the river course noticeably eroded.

**Figure 10:**
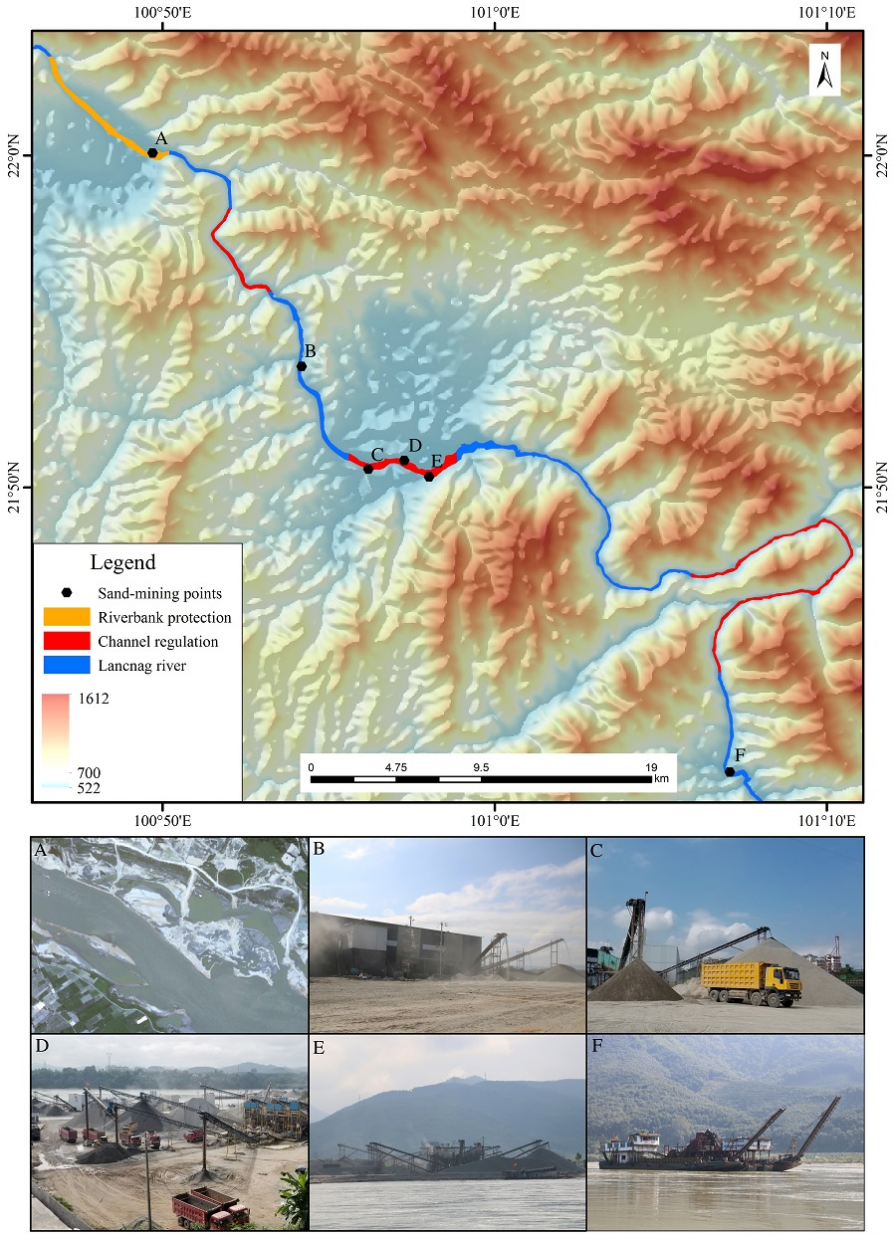
Distribution of sand-mining points in Jinghong to Guanlei reach of the LCR. (A) Jinghong sand-mining points which was stopped after 2008; (B) sand-mining in the thirdbranch of Ganlanba factory; (C), (D) and (E) are tree sand-mining points in Ganlanba reach; (F) sand-minting point in Guanlei reach. The Digital Elevation Model (DEM) in Fig. 10 was provided by the Data Center for Resources and Environmental Sciences, Chinese Academy of Science (http://www.resdc.cn/).

### Impact of river-bed mining

Sediment on both sides of Lancang-Mekong has long been an important source of sand for local infrastructure construction (*Anthony, Brunier & Besset, 2015*. According to the field survey from November 24 to 28, 2018 and images comparison, we detected six sand-mining points downstream of Jinghong to Guanlei reach,with three in GLB, two large ones in Guanlei and a large one in Jinghong which ceased operation since 2008 (Fig. 10). Sand-mining changes the planforms and composition of local river beds, and destroys the balance of sediment transport. Sand-mining on the Jinghong to Guanlei reach of LCR affected mainly four river parameters: riverbed sediment composition, riverbed planforms, flow direction, and sediment transport characteristics.

First, sand-mining intensifies the scouring and widening of river bed and the adjustment of stream gradient. In the Gaozhuang reach of JHB, the riverbed has been deepened and widened, the shoal disappeared, and the rapids became slower. Second, sand-mining destabilizes the conditions in the channel. The beach at GLB ferry terminal is located on the convex bank, and the riverbed shape is relatively stable under natural conditions. However, in the beginning of 2010, large-scale sand-mining activities decreased the elevation, and the stability between the beach and channel was broken. The river mainstream line is northward and beach erosion is evident. Third, sand-mining tends to remove coarse and reserve fine sand particles, leading to sand coarsening. In JHB and GLB reach, riverbed particles have become evidently coarser over time. Finally, sand-mining changes the direction of local water and sediment, as well as characteristics of channel sediment transport. Thus, the river morphology changed due to sand excavation in Manting Sand Bar of GLB. When the sediment load from the upstream decreased, the cross-reach of the downstream sediment excavation pit was difficult to recover, the channel was heavily disturbed, and the river was scoured and eroded.

Due to the construction of cascade reservoirs in the middle and upper reaches of LCR, the sediment load in the lower reach of LCR decreased significantly (Liu et al., 2013; Liu, Trinder & Turner, 2017; Truong, Ye & Stive, 2017). As a result, sand pits remaining from sand-mining in the lower reaches cannot be filled due to insufficient sediment supply, leading to further changes in river morphology in typical river reaches such as JHB and GLB. Therefore, river sand-mining plays an important role in river morphology adjustment to balance in the wide valley reach of lower reaches of LCR.

## Conclusions

Except for the broad-valley reach in GLB and JHB, changes in river morphology in the lower reaches of LCR from Jinghong to Guanlei were not significant. The broad valley reach mainly features river erosion and disappearance of a branch river. During the period of 1993 to 2016, JHB reach in lower LCR was mainly eroded, with an erosion area of 0.36 km^2^. Erosion on the right bank was more evident than that on the left bank. The area of instantaneous water surface and of central bar decreased, while beach area increased. Erosion and accretion areas in GLB were 0.33 km^2^ and 0.61 km^2^ respectively, and the right bank was dominated by erosion, while the left bank by accretion. Additionally, the instantaneous water surface area and beach area increased, while the area of central shoal decreased. Morphological changes in the lower LCR were mainly affected by cascade hydropower development, river regulation, and sand-mining. Cascade hydropower development has significantly changed the runoff sediment relationship downstream, and this was manifested in reductions in flood water level and sediment accretion in the broad valley reach. River regulation has significantly reduced the flood water level in GLB reach, and river bank protection projects have artificially restricted erosion of the watercourse in the upper reach in JHB; large-scale sand mining accelerates changes in river morphology in the typical reach when sand amounts are reduced upstream.

##  Supplemental Information

10.7717/peerj.9471/supp-1 Supplemental Information 1 Raw data

## Additional Information and Declarations

### Data Availability

The following information was supplied regarding data availability: The raw measurements are available as a Supplementary File.
